# Supplementary material for: Mathematical modeling of Dengue virus serotypes propagation in Mexico
Source: PLoS One. 2023 Jul 14;18(7):e0288392. doi: 10.1371/journal.pone.0288392 (PMC10348539; doi:10.1371/journal.pone.0288392)
Supplement: S1 File — (DOCX) [file pone.0288392.s001.docx]

**Code of the program**

The following program is the one used to solve the differential equation system used in our article. The code is compatible with Wolfran´s Mathematica 8 software or latter version:

SimYears=18;

loops=SimYears*12*30;

TempOpt1=22;

TempOpt2=28;

InitialYear=1994;

CasosDat={38000,32000,55000,24000,23000,2000,5000,16000,7000,8000,22000,30000,53000,47000,57000,29000,16000,33000,62000,32000,27000,18000,12000,9000};

CasosHemoDat={500,500,1000,500,100,10,50,1500,1200,1150,4500,5000,9000,7500,12000,6000,4000,18000,19000,8500,5500,3500,500,1000};

SeroObs={{0.18,0.38,0.12,0.33},{0.28,0.03,0.59,0.09},{0.06,0.02,0.87,0.05},{0.05,0.02,0.95,0.0},{0.04,0.14,0.44,0.02},{0.02,0.57,0.22,0.01},{0.01,0.79,0.19,0.0},{0.02,0.82,0.17,0.01},{0.04,0.83,0.08,0.04},{0.04,0.87,0.08,0.05},{0.13,0.79,0.08,0.01},{0.33,0.18,0.4,0.1},{0.91,0.4,0.08,0.0}};

HealthAccess=0.2;

Sint1=0.42;

Sint2=0.08;

Sint3=0.35;

Sint4=0.1;

Hemo1=0.18;

Hemo2=0.19;

Hemo3=0.03;

Hemo4=0.18;

(*Auxiliar variables*)

Sol1={};

Sol2={};

Sol3={};

Sol4={};

SolTemp={};

SolPreci={};

Temperature={};

Rain={};

(*Demographic and epidemiological data*)

H=3.9;(*Persons per house*)

TempHouse=23;(*Minimal inhouse temperature*)

RainCut=1;(*Rainfall cutoff*)

(*Introduction of meteorological data*)

L=WeatherData["Merida", "MeanTemperature", {{InitialYear, 1,1}, {InitialYear+SimYears, 12, 31}, "Month"}];

P=WeatherData["Merida", "TotalPrecipitation", {{InitialYear, 1, 1}, {InitialYear+SimYears, 12, 31}, "Month"}];

(*Introducing meteorological data into vectors*)

For[n=1,n<Length[L]+1,n++,

a=Extract[L,{n,2}];

If[a¹Missing["NotAvailable"],Continue,a=Extract[L,{n-12,2}]];

If[a¹Missing["NotAvailable"],Continue,a=Extract[L,{n+12,2}]];

Temperature=Join[Temperature,{a}];

a=Extract[P,{n,2}];

If[a¹Missing["NotAvailable"],Continue,a=Extract[P,{n-12,2}]];

If[a¹Missing["NotAvailable"],Continue,a=Extract[P,{n+12,2}]];

Rain=Join[Rain,{a}];

];

PluProm=Mean[Rain];

TempMax=Mean[Temperature];

Datos1=Table[Temperature[[n*12+m]],{n,0,SimYears},{m,1,12}];

Datos2=Table[Rain[[n*12+m]],{n,0,SimYears},{m,1,12}];

Minimos={};

Maximos1={};

Maximos2={};

For[n=0,n<SimYears,n++,

resul1=Min[Datos1[[n+1]]];

resul2=Max[Datos1[[n+1]]];

resul3=Max[Datos2[[n+1]]];

Minimos=Join[Minimos,{{n*365+0.5*30.4,resul1}}];

Maximos1=Join[Maximos1,{{n*365+5*30.4,resul2}}];

Maximos2=Join[Maximos2,{{n*365+5.5*30.4,resul3}}];

If[nŠ(SimYears-1),Minimos=Join[Minimos,{{(n+1)*365+0.5*30.4,resul1}}],Continue];

]

resul1=Fit[Minimos,Table[t^n,{n,0,IntegerPart[SimYears/1.5]}],t];

resul2=Fit[Maximos1,Table[t^n,{n,0,IntegerPart[SimYears/1.5]}],t];

resul3=Fit[Maximos2,Table[t^n,{n,0,IntegerPart[SimYears/1.5]}],t];

Preci=1.5+resul3*Exp[-0.0000001*((20000*Sin[(t-30)/365 Pi+80]^2)/2)^2^];

Temp=resul1+(resul2-resul1)*Sin[t/365 Pi]^2^;

(*Deffinig inhouse temperature*)

TempIn=Temp-(1.3^(Temp-TempHouse)/2^-1);

(*variable polynomial deffinition*)

Surb=-90.664+9.54TempIn-0.1855TempIn^2^;

EggsNumb=-71.066+7.597 Temp-0.1428 Temp^2^;

TiempoHuevo=37.066-2.0853Temp+0.0319 Temp^2^;

TiempoLarva=55.499-2.8617 Temp+0.0411 Temp^2^;

TiempoPupa=18.786-1.006 Temp+0.0148 Temp^2^;

(*Variable deffinitions*)

k_2_=1/TiempoHuevo;

k_3_=0.38/TiempoHuevo;

k_4_=1/TiempoLarva;

k_5_=d 0.25/TiempoLarva;

k_6_=0.05;

k_7_=1/TiempoPupa;

k_8_=0.09k_7_;

k_10_=1/Surb;

k_11_=1.56*k_10_;

k_12_=0.6*0.33*2.1*0.6*k_11_*0.5;

k_1_=k_12_EggsNumb/2;

k_14_=1/14;

k_15_=0.00000065;

k_18_=UnitStep[Preci-RainCut];

k_17_=(1-k_18_);

k_13_=0.99*k_17_;

k_19_=0.018;

d=1-(0.1389-0.0136*Preci);

m=0.01;

t=1/(7414Exp[-0.241*TempIn]);

b=0.9;

a=1/(6*12*30);

PrevIniHum=0.3*H;

PrevIniMos=0.03;

InfIniHum=0.12*H;

k_9,1_=0.67*4*0.8; (*Comparative Susceptibility of Aedes albopictus and Aedes aegypti to Dengue Virus Infection After Feeding on Blood of Viremic Humans:Implications for Public Health*)

k_9,2_=k_9,1_;(*Duration of Dengue Viremia in Blood Donors and Relationships Between Donor Viremia,Infection Incidence and Clinical Case Reports During a Large Epidemic.*)

k_9,3_=k_9,1_;

k_9,4_=k_9,1_;(*infeccion humano-mosquito*)

k_16,1_=0.9a;

k_16,2_=1a;

k_16,3_=0.45 a;

k_16,4_=0.07 a;

Inf1=x_5_[t]+x_6_[t]+(1-2k_16,2_)y_6_[t]+(1-2k_16,3_)z_6_[t]+(1-2k_16,4_)w_6_[t];

(*los que no se pueden infectar, Co-circulation and co-infections of all dengue virus serotypes in Hyderabad,India 2014*)

Inf2=y_5_[t]+y_6_[t]+(1-2k_16,1_)x_6_[t]+(1-2k_16,3_)z_6_[t]+(1-2k_16,4_)w_6_[t];

Inf3=z_5_[t]+z_6_[t]+(1-2k_16,1_)x_6_[t]+(1-2k_16,2_)y_6_[t]+(1-2k_16,4_)w_6_[t];

Inf4=w_5_[t]+w_6_[t]+(1-2k_16,1_)x_6_[t]+(1-2k_16,2_)y_6_[t]+(1-2k_16,3_)z_6_[t];

T=x_5_[t]+y_5_[t]+z_5_[t]+w_5_[t]+x_6_[t]+y_6_[t]+z_6_[t]+w_6_[t];

S1=s_1_'[t]Šk_1_ (s_4_[t]+(1-m) (x_4_[t]+y_4_[t]+z_4_[t]+w_4_[t]))+k_18_s_5_[t]-(k_2_+k_17_+k_3_) s_1_[t];(*Susceptible eggs*)

S2=s_2_'[t]Šk_2_ s_1_[t]-(k_4_+k_5_+k_6_ s_2_[t]) s_2_[t];(*Susceptible larva*)

S3=s_3_'[t]Šk_4_ s_2_[t]-(k_7_+k_8_) s_3_[t];(*Susceptible pupa*)

S4=s_4_'[t]Šk_7_s_3_[t]-(k_9,1_x_5_[t]+k_9,2_y_5_[t]+k_9,3_z_5_[t]+k_9,4_w_5_[t]+k_10_)s_4_[t];(*Susceptible mosquito*)

S5=s_5_'[t]Šk_17_ s_1_[t]-(k_18_+ k_19_)s_5_[t];(*Resting Susceptible eggs*)

X1=x_1_'[t]Šm k_1_ x_4_[t]+k_18_x_7_[t]-(k_2_+k_17_+k_3_) x_1_[t];(*Infected eggs*)

X2=x_2_'[t]Šk_2_ x_1_[t]-(k_4_+k_5_+k_6_ x_2_[t]) x_2_[t];(*Infected larva*)

X3=x_3_'[t]Šk_4_ x_2_[t]-(k_7_+k_8_) x_3_[t];(*Infected pupa*)

X4=x_4_'[t]Šk_7_ x_3_[t]+k_9,1_ x_5_[t] s_4_[t]-k_11_ x_4_[t];(*Infected mosquito*)

X5=x_5_'[t]Št k_12_ (H-Inf1)x_4_[t](1-b (x_5_[t]+x_6_[t])/T)-(k_13_+k_14_) x_5_[t];(*Infected human*)

X6=x_6_'[t]Šk_14_ x_5_[t]-(k_16,1_+k_15_) x_6_[t];(*Immune human*)

X7=x_7_'[t]Šk_17_ x_1_[t]-(k_18_+k_19_)x_7_[t];(*Resting infected eggs*)

Y1=y_1_'[t]Šm k_1_ y_4_[t]+k_18_y_7_[t]-(k_2_+k_17_+k_3_) y_1_[t];(*Infected eggs*)

Y2=y_2_'[t]Šk_2_ y_1_[t]-(k_4_+k_5_+k_6_ y_2_[t]) y_2_[t];(*Infected larva*)

Y3=y_3_'[t]Šk_4_ y_2_[t]-(k_7_+k_8_) y_3_[t];(*Infected pupa*)

Y4=y_4_'[t]Šk_7_ y_3_[t]+k_9,2_ y_5_[t] s_4_[t]-k_11_ y_4_[t];(*Infected mosquito*)

Y5=y_5_'[t]Št k_12_(H-Inf2)y_4_[t](1-b (y_5_[t]+y_6_[t])/T)-(k_13_+k_14_) y_5_[t];(*Infected human*)

Y6=y_6_'[t]Šk_14_ y_5_[t]-(k_16,2_+k_15_) y_6_[t];(*Immune human*)

Y7=y_7_'[t]Šk_17_ y_1_[t]-(k_18_+k_19_)y_7_[t];(*Resting infected eggs*)

Z1=z_1_'[t]Šm k_1_ z_4_[t]+k_18_z_7_[t]-(k_2_+k_17_+k_3_) z_1_[t];(*Infected eggs*)

Z2=z_2_'[t]Šk_2_ z_1_[t]-(k_4_+k_5_+k_6_ z_2_[t]) z_2_[t];(*Infected larva*)

Z3=z_3_'[t]Šk_4_ z_2_[t]-(k_7_+k_8_) z_3_[t];(*Infected pupa*)

Z4=z_4_'[t]Šk_7_ z_3_[t]+k_9,3_ z_5_[t] s_4_[t]-k_11_ z_4_[t];(*Infected mosquito*)

Z5=z_5_'[t]Št k_12_(H-Inf3)z_4_[t](1-b (z_5_[t]+z_6_[t])/T)-(k_13_+k_14_) z_5_[t];(*Infected human*)

Z6=z_6_'[t]Šk_14_ z_5_[t]-(k_16,3_+k_15_) z_6_[t];(*Immune human*)

Z7=z_7_'[t]Šk_17_ z_1_[t]-(k_18_+k_19_)z_7_[t];(*Resting infected eggs*)

W1=w_1_'[t]Šm k_1_ w_4_[t]+k_18_w_7_[t]-(k_2_+k_17_+k_3_) w_1_[t];(*Infected eggs*)

W2=w_2_'[t]Šk_2_ w_1_[t]-(k_4_+k_5_+k_6_ w_2_[t]) w_2_[t];(*Infected larva*)

W3=w_3_'[t]Šk_4_ w_2_[t]-(k_7_+k_8_) w_3_[t];(*Infected pupa*)

W4=w_4_'[t]Šk_7_ w_3_[t]+k_9,4_ w_5_[t] s_4_[t]-k_11_ w_4_[t];(*Infected mosquito*)

W5=w_5_'[t]Št k_12_(H-Inf4)w_4_[t](1-b (w_5_[t]+w_6_[t])/T)-(k_13_+k_14_) w_5_[t];(*Infected human*)

W6=w_6_'[t]Šk_14_ w_5_[t]-(k_16,4_+k_15_) w_6_[t];(*Immune human*)

W7=w_7_'[t]Šk_17_ w_1_[t]-(k_18_+k_19_)w_7_[t];(*Resting infected eggs*)

Frac1=0.6;

Frac2=0.2;

Frac3=0.003;

Frac4=1-(Frac1+Frac2+Frac3);

Pop=800000*(1+0.0000547*t)/H;

sol=NDSolve[{S1,S2,S3,S4,S5,X1,X2,X3,X4,X5,X6,X7,Y1,Y2,Y3,Y4,Y5,Y6,Y7,Z1,Z2,Z3,Z4,Z5,Z6,Z7,W1,W2,W3,W4,W5,W6,W7,

s_1_[0]Š0,s_2_[0]Š0,s_3_[0]Š0,s_4_[0]Š1-PrevIniMos,s_5_[0]Š0,

x_1_[0]Š0,x_2_[0]Š0,x_3_[0]Š0,x_4_[0]ŠFrac1*PrevIniMos,x_5_[0]ŠFrac1*InfIniHum,x_6_[0]ŠFrac1*PrevIniHum,x_7_[0]Š0,

y_1_[0]Š0,y_2_[0]Š0,y_3_[0]Š0,y_4_[0]ŠFrac2*PrevIniMos,y_5_[0]ŠFrac2*InfIniHum,y_6_[0]ŠFrac2*PrevIniHum,y_7_[0]Š0,

z_1_[0]Š0,z_2_[0]Š0,z_3_[0]Š0,z_4_[0]ŠFrac3*PrevIniMos,z_5_[0]ŠFrac3*InfIniHum,z_6_[0]ŠFrac3*PrevIniHum,z_7_[0]Š0,

w_1_[0]Š0,w_2_[0]Š0,w_3_[0]Š0,w_4_[0]ŠFrac4*PrevIniMos,w_5_[0]ŠFrac4*InfIniHum,w_6_[0]ŠFrac4*PrevIniHum,w_7_[0]Š0},

{s_1_,s_2_,s_3_,s_4_,s_5_,x_1_,x_2_,x_3_,x_4_,x_5_,x_6_,x_7_,y_1_,y_2_,y_3_,y_4_,y_5_,y_6_,y_7_,z_1_,z_2_,z_3_,z_4_,z_5_,z_6_,z_7_,w_1_,w_2_,w_3_,w_4_,w_5_,w_6_,w_7_},{t,0,loops}];

For[n=12*30,n<loops,n++,

Sol1=Join[Sol1,{Evaluate[x_5_[n]/.sol]}[[1]]];

Sol2=Join[Sol2,{Evaluate[y_5_[n]/.sol]}[[1]]];

Sol3=Join[Sol3,{Evaluate[z_5_[n]/.sol]}[[1]]];

Sol4=Join[Sol4,{Evaluate[w_5_[n]/.sol]}[[1]]];

]

For[n=0,n<(SimYears*12*4),n++,

TempA=Temperature[[IntegerPart[n/4]+1]];

PreciA=Rain[[IntegerPart[n/4]+1]];

SolTemp=Join[SolTemp,{{n*7.6,TempA}}];

SolPreci=Join[SolPreci,{{n*7.6,PreciA}}];

]

Acum1=Table[{Sum[Sol1[[m]],{m,n*12*30+1,(n+1)*12*30}],Sum[Sol2[[m]],{m,n*12*30+1,(n+1)*12*30}],Sum[Sol3[[m]],{m,n*12*30+1,(n+1)*12*30}],Sum[Sol4[[m]],{m,n*12*30+1,(n+1)*12*30}]},{n,0,SimYears-2}];

Deno=Table[{Acum1[[n]][[1]]+Acum1[[n]][[2]]+Acum1[[n]][[3]]+Acum1[[n]][[4]]}[[1]],{n,1,SimYears-1}];

Data1=Table[{Acum1[[n]][[1]]/Deno[[n]],Acum1[[n]][[2]]/Deno[[n]],Acum1[[n]][[3]]/Deno[[n]],Acum1[[n]][[4]]/Deno[[n]]},{n,1,SimYears-1}];

Data2=Table[(Acum1[[n]][[1]]*Sint1+Acum1[[n]][[2]]*Sint2+Acum1[[n]][[3]]*Sint3+Acum1[[n]][[4]]*Sint4)*1000000*(1+0.0000547*n)/H*HealthAccess,{n,1,SimYears-1}];

Data3=Table[(Acum1[[n]][[1]]*Sint1*Hemo1+Acum1[[n]][[2]]*Sint2*Hemo2+Acum1[[n]][[3]]*Sint3*Hemo3+Acum1[[n]][[4]]*Sint4*Hemo4)*1000000*(1+0.0000547*n)/H*HealthAccess,{n,1,SimYears-1}];

(*Graphs*)

B1A=Plot[{Evaluate[Temp/.sol],Evaluate[TempIn/.sol]},{t,0,loops},PlotStyle®{{Directive[Black],Thickness[0.005]},{Directive[Red],Thickness[0.005]}},Frame®True,FrameTicksStyle®Directive[16],PlotRange®{20,32},FrameLabel®{Style["Time (Years)",FontSize®18],Style["Temperature (°C)",FontSize®18]},Axes®False,FrameTicks®{{Automatic,None},{Table[{n*12*30,ToString[InitialYear+n+1]},{n,0,SimYears,5}],None}}];

B2A=Plot[Evaluate[Preci/.sol],{t,0,loops},PlotStyle®{Directive[Black],Thickness[0.005]},Frame®True,FrameTicksStyle®Directive[16],PlotRange®{0,70},FrameLabel®{Style["Time (Years)",FontSize®18],Style["Precipitation (mm of rain)",FontSize®18]},Axes®False,FrameTicksStyle®Directive[16],FrameTicks®{{Automatic,None},{Table[{n*12*30,ToString[InitialYear+n+1]},{n,0,SimYears,5}],None}}];

B1B=ListLinePlot[SolTemp,PlotStyle®{Directive[Blue]},Frame®True,FrameTicksStyle®Directive[16],PlotRange®{15,30},Axes®False];

B2B=ListLinePlot[SolPreci,PlotStyle®{Directive[Blue]},Frame®True,FrameTicksStyle®Directive[16],PlotRange®{0,70},Axes®False];

B1=Show[B1A,B1B];

B2=Show[B2A,B2B];

B3=Plot[{Evaluate[x_5_[t]/(H/100000)*HealthAccess*Sint1/.sol],Evaluate[y_5_[t]/(H/100000)*HealthAccess*Sint2/.sol],Evaluate[z_5_[t]/(H/100000)*HealthAccess*Sint3/.sol],Evaluate[w_5_[t]/(H/100000)*HealthAccess*Sint4/.sol]},{t,12*30,loops},PlotStyle®{Directive[Black],Directive[Red],Directive[Blue],Directive[Green]},Frame®True,FrameTicksStyle®Directive[16],PlotRange®{0,60},FrameLabel®{Style["Time (Years)",FontSize®18],Style["Incidence (per 100 000 persons)",FontSize®18]},Axes®False,FrameTicks®{{Automatic,None},{Table[{n*12*30,ToString[InitialYear+n-1]},{n,0,SimYears,5}],None}}];

B4=Plot[{Evaluate[(x_5_[t]+x_6_[t])*HealthAccess*Sint1/.sol],Evaluate[(y_5_[t]+y_6_[t])*HealthAccess*Sint2/.sol],Evaluate[(z_5_[t]+z_6_[t])*HealthAccess*Sint3/.sol],Evaluate[(w_5_[t]+w_6_[t])*HealthAccess*Sint4/.sol]},{t,12*30,loops},PlotStyle®{Directive[Black],Directive[Red],Directive[Blue],Directive[Green]},Frame®True,FrameTicksStyle®Directive[16],PlotRange®Automatic,FrameLabel®{Style["Time (Years)",FontSize®18],Style["Prevalence",FontSize®18]},Axes®False,FrameTicks®{{Automatic,None},{Table[{n*12*30,ToString[InitialYear+n+1]},{n,0,SimYears,5}],None}}];

Casos=Table[{InitialYear+n,CasosDat[[n]]+CasosHemoDat[[n]]},{n,1,SimYears-2}];

CasosHemo=Table[{InitialYear+n,CasosHemoDat[[n]]},{n,1,SimYears-2}];

CasosMod=Table[{InitialYear+n,Data2[[n]]},{n,1,SimYears-1}];

CasosHemoMod=Table[{InitialYear+n,Data3[[n]]},{n,1,SimYears-1}];

Den1Mod=Table[{InitialYear+n,Data1[[n]][[1]]},{n,1,Length[Data1]}];

Den2Mod=Table[{InitialYear+n,Data1[[n]][[2]]},{n,1,Length[Data1]}];

Den3Mod=Table[{InitialYear+n,Data1[[n]][[3]]},{n,1,Length[Data1]}];

Den4Mod=Table[{InitialYear+n,Data1[[n]][[4]]},{n,1,Length[Data1]}];

Den1=Table[{InitialYear+n,SeroObs[[n]][[1]]},{n,1,Length[SeroObs]}];

Den2=Table[{InitialYear+n,SeroObs[[n]][[2]]},{n,1,Length[SeroObs]}];

Den3=Table[{InitialYear+n,SeroObs[[n]][[3]]},{n,1,Length[SeroObs]}];

Den4=Table[{InitialYear+n,SeroObs[[n]][[4]]},{n,1,Length[SeroObs]}];

B5=ListLinePlot[{Den1,Den2,Den3,Den4,Den1Mod,Den2Mod,Den3Mod,Den4Mod},PlotStyle®{{Directive[Black],Dashed},{Directive[Red],Dashed},{Directive[Blue],Dashed},{Directive[Green],Dashed},{Directive[Black],Thickness[0.005]},{Directive[Red],Thickness[0.005]},{Directive[Blue],Thickness[0.005]},{Directive[Green],Thickness[0.005]}},Frame®True,FrameTicksStyle®Directive[16],PlotRange®{0,1},Axes®False];

B6=ListLinePlot[{Casos,CasosHemo,CasosMod,CasosHemoMod},PlotStyle®{{Directive[Orange],Dashed},{Directive[Red],Dashed},{Directive[Orange],Thickness[0.005]},{Directive[Red],Thickness[0.005]}},Frame®True,FrameTicksStyle®Directive[16],PlotRange®{0,80000},Axes®False];

GraphicsGrid[{{B1,B2},{B3,B4},{B5,B6}}]
