# Supplementary material for: Mathematical modeling of Dengue virus serotypes propagation in Mexico
Source: PLoS One. 2023 Jul 14;18(7):e0288392. doi: 10.1371/journal.pone.0288392 (PMC10348539; doi:10.1371/journal.pone.0288392)
Supplement: Attachment — Submitted filename: Response to Reviewers.docx [file pone.0288392.s003.docx]

1.	Some specific background to the dengue burden and epidemiology in Mexico would be useful in the introduction, particularly with reference to some of the findings from earlier publications later used as model parameters (e.g. DENV1-4 prevalence over time).

Resp: We introduced the next paragraph in the introduction section:

“The south-southeast regions of Mexico are endemic for dengue, showing incidence variations depending on the specific weather condition. The magnitude of the epidemic outbreaks also depends on the circulating serotypes. As a consequence, a dengue epidemiological surveillance of the 32 states of the Mexican Republic is performed by the General Directorate of Epidemiology (DGE) [6]. Even though prevention and vector control efforts have been ramped up, the Dengue cases have been multiplied by ten during the last 20 years, reaching 264,898 cases in 2019 [6]. It is worth mentioning that it has been stated that underreporting and misdiagnosis of the DENV cases limit the ability to determine the true burden of disease [7].”

2.	In line 14 the authors state that they “used a mathematical model to determine the outbreak probability of specific serotypes of DENV” as no outbreak probabilities are calculated in this paper I would suggest making the aims clearer and more related to the final analysis (for instance, ‘predicting serotype-specific DENV prevalence and overall case burden in Mexico’).

Resp: thank you very much for the suggestion, we changed the sentence to: “Here, we used a mathematical model to predict serotype-specific DENV prevalence and overall case burden in Mexico.”

3.	Methods: the model equations in the revised text are much clearer.

- Parameter beta is described as a calibration parameter - some description of how calibration was performed would be useful in the main text.

Resp: we included the following mention in the methods section: “β is a calibration parameter, which serves for finding a suitable value (weight) for the fraction of the infected persons of the former serotype, with respect to all serotypes, in the cut-off function ϴ. The value of β was not determined by any optimization algorithm but through a try and failure way.”

- The revised equations on lines 133 - 147 are substantially clearer. To improve the readability further it would be useful if a consistent subscript was used for parameters which are serotype-dependent.

Resp: only the parameters k_16 are serotype-dependent, and they carry a subscript which define the serotypes referred to.

- It would be useful to define theta in the parameter table as well as the main text.

Resp: Good observation, the theta of the table is the Heaviside Theta, which is now clarified in the text.

- In the parameter table I believe P(t) is used to refer to precipitation - I would recommend altering this to read Precipitation(t) or similar to avoid confusion with probability.

Resp: we changed the name of the variable for R(t) to keep in mind that it refers to rainfall.

- The terminology ‘mosquito emergence deactivation’ and ‘mosquito emergence activation’ is not very clear, I would recommend renaming these perhaps to ‘rate of mosquito emergence’ and ‘rate of mosquito emergence suppression by drought’ or similar.

Resp: we will use the term: Drought induced egg eclosion inhibition and Rain induced egg eclosion rate.

- I think there is an error in the parameter table for the proportion of initial serotypes for DENV 1,2,3 and 4 as the value for DEV 4 reads 19.997. Should this be 0.19997? If not I’m unsure why these would not sum to 1. It would also be useful to give some context on the data / literature informing this parameter in the introduction as it is quite important context to interpret the subsequent serotype-specific results.

Resp: Thank you very much, it was a typographic error, the correct number is 0.197.

- In line 162 authors describe how the indoor temperature is modelled with a pull-down factor reducing the peaks, this makes sense but differs from what is shown in Fig 1.A where the indoor temperature (black) shows higher peaks than the outdoor temperature (red)?

Resp: The black line represents the adjusted curve. In the actualized version of the figures we clarify this point.

- It would be useful to add legends to all figures, particularly the serotype specific plots, for ease of interpretation.

Resp: Done

4.	It’s not clear to me how the initial proportion of serotypes shown in Fig 2.C corresponds with the proportion of initial serotypes for DEV1,2,3 and 4 in the parameter table.

Resp: Please consider that the figure is obtained from the normalization of the outcomes, showing the relative fraction of each serotype. More precisely, the graph is showing the normalization on the sum of the variables S_5 +S_6, hence the initial conditions cannot be seen in a strict way. This is now addressed in the text. We introduced the following phrase: “The serotype composition of the prevalence was obtained from the normalization of the outcomes of the sum of the variables S_5 (t)+S_6 (t), which are the variables that contains the information of the total (incident + prevalent) cases, is presented in the Figure 2C”

5.	More detail on the source and limitations of the data streams compared with model output would be useful - some details on the DENV prevalence data from 1995-2007 and the surveillance used would help readers to qualitatively interpret model fit to data. This could perhaps be included in a data sub-section within the Methods.

Resp: The prevalence and serotypes data were originally collected through the “Centro Nacional de Vigilancia Epidemiológica y Control de Enfermedades”, the mexican national agency in charge of tracking the diseases spread in Mexico, as well as determine the opportune action to be had. The original data are the results of sampling of the total DENV cases where the serotype was determined by PCR. Since only a fraction of the total DENV cases detected were serotyped, the authors extrapolate the serotype proportion to the total DENV cases encountered in the endemic region. The authors mention the caveat of this generalization and the possibility of having a heterogeneous sampling in the population. Nevertheless, the sum of the 4 serotypes do follow the trend of the total dengue cases reported, hence we assumed that the reported proportion of each serotypes is accurate.

The data can be found at the Dirección General de Epidemiología, Secretaría de Salud. Panorama Epidemiológico (México). México, DF: DGE/SS, 2008. We introduced the next paragraph in the methodology section: “. In all these works, the original data were collected through the “Centro de Vigilancia Epidemiológica y Control de Enfermedades”, the national agency in charge of tracking the diseases spread in Mexico. From the total probable Dengue cases, 30% were sampled for confirmation purposes, as per normative guidelines. The serotype of the circulating virus was determined through PCR genotypification of 10% of the previously confirmed cases [6]. The specific weight of the data obtained by this method was then extrapolated to the observed cases. This method is the one generally used by the Mexican national disease surveillance system to monitor the burden of Dengue in the population.”

6.	The model is able to qualitatively capture both the serotype-specific and overall case dynamics (shown in Fig 2.C) which is very interesting. The RMSE is also calculated to assess model accuracy. I would be interested in seeing how this compares to some kind of baseline model (perhaps a simple seasonality / climatological model) although this would depend on the time units of the model presented here.

Resp: We addressed your suggestion in the discussion section: “The prediction capacity of our model gives a root-mean-square deviation of 33.9% per year (and 19.8%, dropping the outlier years), in the prediction accuracy of the total infection cases. There are some example of statistical models which depends of meteorological variables that produces more accurate predictions, like the work published by Ling Hii et al. [53], which reports an accuracy of 0.3% in a 16-weeks forecasting, or the model published by Naher et al. [54], which reports an accuracy fit of 10.8% in a 2-year time-series model. Nevertheless, our model predicts the proportions of DENV serotypes as well as the total amount of DENV infection over a 18-year time-lapse, with an accuracy allowing for long-term public health decisions.”

7. It’s still unclear to me the way in which the different serotypes are differentially affected by temperature as mentioned in line 267, some more discussion on this would be helpful.

Resp: We apologize for leaving the statement about the differential serotype outcome in the last version of the manuscript. We deleted this affirmation in this corrected version of the article.

Minor comments:

- The use of the term ‘dispersal’ in the title is confusing as it suggests a spatial analysis, I would recommend different terminology used by authors later on in the text such as dynamics or prevalence.the

Resp: We took your suggestion into account, we changed the title of the article to “Mathematical modeling of Dengue virus serotypes propagation in Mexico”

- In line 5 I think rather than ‘constitute’ the authors mean ‘constitutes’.

Resp: We corrected the point mentioned by the revisors.

- In line 33 authors refer to the dengue vaccine displaying ‘immunity’ but I believe ‘protection’ or ‘efficacy’ would be less confusing.

Resp: We corrected the point mentioned by the revisors.

- In line 164 “were” should be replaced with “where”

Resp: We corrected the point mentioned by the revisors.

- In line 184 the sentence “we graphed the temperature” could be replaced with “we displayed the temperature”

Resp: We corrected the point mentioned by the revisors.

- In line 274 the term “man made action” is unclear and could be replaced by “public health interventions”.

Resp: We corrected the point mentioned by the revisors.
